# Humoral response to catumaxomab correlates with clinical outcome: Results of the pivotal phase II/III study in patients with malignant ascites

**DOI:** 10.1002/ijc.26258

**Published:** 2011-06-23

**Authors:** Marion G Ott, Frederik Marmé, Gerhard Moldenhauer, Horst Lindhofer, Michael Hennig, Rolf Spannagl, Mirko M Essing, Rolf Linke, Diane Seimetz

**Affiliations:** 1 Fresenius Biotech GmbHMunich, Germany; 2 Department of Obstetrics and Gynecology, National Center for Tumor Diseases, University HospitalHeidelberg, Germany; 3 Translational Immunology Unit, German Cancer Research Center, National Center for Tumor DiseasesHeidelberg, Germany; 4 TRION Pharma GmbHMunich, Germany

**Keywords:** catumaxomab, trifunctional antibody, malignant ascites, humoral response, clinical outcome

## Abstract

The trifunctional antibody catumaxomab is a targeted immunotherapy for the intraperitoneal treatment of malignant ascites. In a Phase II/III trial in cancer patients (*n* = 258) with malignant ascites, catumaxomab showed a clear clinical benefit *vs*. paracentesis and had an acceptable safety profile. Human antimouse antibodies (HAMAs), which could be associated with beneficial humoral effects and prolonged survival, may develop against catumaxomab as it is a mouse/rat antibody. This *post hoc* analysis investigated whether there was a correlation between the detection of HAMAs 8 days after the fourth catumaxomab infusion and clinical outcome. HAMA-positive and HAMA-negative patients in the catumaxomab group and patients in the control group were analyzed separately for all three clinical outcome measures (puncture-free survival, time to next puncture and overall survival) and compared to each other. There was a strong correlation between humoral response and clinical outcome: patients who developed HAMAs after catumaxomab showed significant improvement in all three clinical outcome measures *vs*. HAMA-negative patients. In the overall population in HAMA-positive *vs*. HAMA-negative patients, median puncture-free survival was 64 *vs*. 27 days (*p* < 0.0001; HR 0.330), median time to next therapeutic puncture was 104 *vs*. 46 days (*p* = 0.0002; HR 0.307) and median overall survival was 129 *vs*. 64 days (*p* = 0.0003; HR 0.433). Similar differences between HAMA-positive and HAMA-negative patients were seen in the ovarian, nonovarian and gastric cancer subgroups. In conclusion, HAMA development may be a biomarker for catumaxomab response and patients who developed HAMAs sooner derived greater benefit from catumaxomab treatment.

The trifunctional antibody (trAb) catumaxomab (Removab®, Fresenius Biotech GmbH, Munich, Germany) was approved in the European Union in April 2009 for the intraperitoneal (i.p.) treatment of malignant ascites in patients with epithelial cell-adhesion molecule (EpCAM)-positive carcinomas. Catumaxomab is a targeted immunotherapy that is characterized by its binding to three different cell types: tumor cells; T-cells and accessory cells.^1^–^3^ It is composed of mouse and rat immunoglobulin (Ig)G that binds to human EpCAM on tumor cells and human CD3 receptors on T-cells, respectively. The hybrid Fc region of the intact antibody binds preferentially to Type I, IIa and III Fcγ receptors on accessory cells, *e.g*., natural killer cells, dendritic cells and macrophages.^3^, ^4^ The interaction of different immune effector cells at the tumor site induces a complex immune reaction that results in the targeted elimination of tumor cells.^5^ Moreover, trAbs could be shown to induce a long-term humoral antitumor effect.^4^–^7^

As catumaxomab is a nonhumanized chimeric antibody derived from mouse/rat IgG, it is potentially immunogenic when administered to humans. Thus, the development of antidrug antibodies (ADAs) against the murine and rat components of the antibody molecule is to be expected. In clinical studies, a high percentage of patients treated with catumaxomab developed antibodies against mouse [human antimouse antibodies (HAMAs)] and rat [human antirat antibodies (HARAs)] proteins.

The efficacy of catumaxomab in the treatment of malignant ascites was demonstrated in a pivotal, randomized, open-label, Phase II/III trial (EudraCT 2004-000723-15; NCT00836654).^8^ In this study, cancer patients (*n* = 258) with recurrent symptomatic malignant ascites resistant or refractory to conventional chemotherapy were stratified by cancer type (ovarian and nonovarian cancer, 129 patients in each group) and randomized 2:1 to either a single paracentesis followed by i.p. catumaxomab or paracentesis alone (control group). Puncture-free survival, defined as the time to first need for therapeutic puncture or death after treatment, whichever occurred first, was the primary efficacy endpoint; overall survival (OS) and time to next puncture were secondary endpoints.

The addition of catumaxomab resulted in significantly prolonged puncture-free survival and time to next puncture and improved OS.^8^ Puncture-free survival and time to next puncture were both significantly (*p* < 0.0001) prolonged by paracentesis plus catumaxomab *vs*. paracentesis alone in the overall population, the ovarian and nonovarian cancer populations and in patients with gastric cancer, the largest subgroup of nonovarian cancer patients. In the overall population, median puncture-free survival was 46 *vs*. 11 days [*p* < 0.0001; hazard ratio (HR) 0.254] and median time to next puncture was 77 *vs*. 13 days (*p* < 0.0001; HR 0.169). OS showed a positive trend for paracentesis plus catumaxomab in the overall (1-year survival rate 10.4% *vs*. 3.4%; *p* = 0.0846; HR 0.723), ovarian cancer (1-year survival rate 19.7% *vs*. 9.0%; *p* = 0.1543; HR 0.650) and nonovarian cancer (6-month survival rate 17.2% *vs*. 4.9%; *p* = 0.4226; HR 0.825) populations and was significantly prolonged in patients with gastric cancer (6-month survival rate 17.3% *vs*. 0% and median OS 71 *vs*. 44 days; *p* = 0.0313; HR 0.469).

As catumaxomab is a mouse/rat antibody, HAMAs against catumaxomab may develop. The development of HAMAs in response to the administration of murine antibodies is a well-recognized phenomenon and is not associated with any major safety concerns.^9^–^11^ In addition, the development of HAMAs may be associated with beneficial humoral effects and prolonged survival.^11^–^17^ Lanzavecchia *et al*. showed that, in patients treated with a mouse monoclonal antibody (mAb), the mAb generated T-cells that were specific for the mouse immunoglobulin and were able to recognize and in some cases kill, target cells that had bound the mAb.^16^ In a study of ovarian cancer patients treated with the bispecific antibody F(ab)2 OC/TR, increased HAMA levels after treatment were associated with longer median survival.^17^ In the pivotal Phase II/III study in malignant ascites, as expected, most patients developed HAMAs against catumaxomab.^8^

The objective of this *post hoc* analysis was to investigate whether there was a correlation between the humoral response, as measured by HAMA status 8 days after completion of catumaxomab treatment and clinical outcome in patients with malignant ascites. This is the first time this potential correlation between humoral response and clinical outcome has been investigated in a pivotal trial of this nature.

## Material and Methods

### Study design

The pivotal study was a two-arm, randomized, open-label, Phase II/III trial in patients with symptomatic malignant ascites secondary to epithelial cancers requiring paracentesis. The study (EudraCT number: 2004-000723-15; ClinicalTrials.gov identifier: NCT00836654) was approved by an independent ethics committee at each study center and all patients gave written informed consent before participation. The study was conducted in compliance with Good Clinical Practice guidelines and the Declaration of Helsinki. Patients were randomized 2:1 to paracentesis plus catumaxomab (catumaxomab group) or paracentesis alone (control group) and stratified by cancer type (ovarian or nonovarian). Catumaxomab was administered as four infusions of 10, 20, 50 and 150 μg on days 0, 3, 7 and 10, respectively, via an i.p. catheter. Before each catumaxomab infusion and 1 day after the last infusion, the remaining fluid was drained from the peritoneal cavity via the indwelling catheter. The control group received one therapeutic paracentesis only on Day 0. In both groups, repuncture was performed if patients required relief of ascites symptoms.

Patients were assessed at 8 days (Visit 6) and 1, 3, 5 and 7 months (end of study) after the last infusion (catumaxomab group) or after the initial therapeutic paracentesis (Day 0, control group). The end of the study was reached when the patient required the next paracentesis or died, whichever occurred first. After reaching the primary endpoint, all patients were further assessed every 2 months until death or 6 months after the last patient was randomized, whichever was later, for the evaluation of OS. Patients in the control group who fulfilled the eligibility criteria and had two therapeutic punctures after Day 0 were permitted to receive catumaxomab in a subsequent, single-arm, crossover period.

### Detection of HAMA

For patients treated with catumaxomab, HAMAs were analyzed in serum samples at screening, before the third infusion, before the fourth infusion, 8 days and 1 month after the last infusion and at puncture visit. A commercially available *in vitro* diagnostic test, HAMA-ELISA medac (medac GmbH, Hamburg, Germany), a simple and rapid one-step enzyme immunoassay for the quantitative determination of HAMAs in serum, was used. The test was performed according to the manufacturer's instructions and calibrated against goat antimouse IgG antibodies, with a measuring range of 40–2000 ng/mL. Briefly, microtiter plates were precoated with mouse IgG and bovine serum albumin. Clinical-trial samples and peroxidase-labeled mouse IgG (conjugate) were then added. HAMAs bind to the solid phase and peroxidase-labeled mouse IgG. For detection of bound HAMAs, the substrate for the enzyme reaction with H_2_O_2_ was 3,3′,5,5′-tetramethylbenzidine. The reaction was stopped by the addition of sulfuric acid. The absorption of the colored product was measured photometrically at 450 nm (reference wavelength: 620–650 nm). Calibration was performed by polyclonal goat antimouse IgG. Internal tests to detect anticatumaxomab antibodies, HAMAs and human antirat antibodies showed significant correlations with the results of the Medac test. Thus, the Medac HAMA test is an appropriate method for the determination of anticatumaxomab antibodies. A threshold of 40 ng/mL for the determination of a positive or negative HAMA was used according to the manufacturer's instructions (Medac, Hamburg, Germany).

The in-house HAMA and HARA assays were sandwich enzyme-linked immunosorbent assays (ELISAs) performed in a 96-well microtiter plate (MaxiSorp™, Nunc GmbH, KG, Langenselbold, Germany). A mixture of mouse and rat Ig as capture antibodies (4 μg/mL each in 100 mM sodium, hydrogen, carbonate buffer pH 8.0) was added to each well for 15–24 hours at 15–25°C. After washing three times with water, bovine serum albumin (BSA) solution (10 mg/mL BSA and 0.2 mL/L Kathon in 0.2 M Tris-HCl pH 7.5) was added for 15–24 hrs at 15–25°C to saturate residual protein binding capacity. Four different horseradish-peroxidase-labeled monoclonal detection antibodies were used in separate assays: mouse Ig; mouse IgG2a; rat Ig and rat IgG2b.

Serum sample response calibrator (100 μL diluted in 0.1 mM sodium phosphate pH 7.5, 200 mL/L heat-inactivated fetal calf serum, 1 mL/L phenol and 0.4 mL/L Kathon) and 25 μL of the respective detection antibody solutions were added. Each sample was tested by the addition of an excess of mouse and rat Ig that inhibits the specific signal (confirmation assay). After incubation for 16–24 hrs at 15–25°C, the plate was washed at least six times with wash solution (20 mM sodium phosphate pH 7.5, 13 mM sodium chloride, 0.5 mL/L Tween 20 and 0.1 mL Kathon).

The substrate solution for the enzyme reaction was 0.5 mM 3,3′,5,5′-tetramethylbenzidine, 5 mL/L acetone, 45 mL/L ethanol, 4 mM H_2_O_2_ and 0.15 mL/L Kathon in 30 mM sodium citrate buffer pH 4.1. The reaction was stopped by the addition of 1 M sulphuric acid and the absorbance was measured at 450 nm. The difference in the sample signal to the confirmation signal (representing unspecific binding of the detection antibodies to the microtiter plate) was used for quantification. Calibration was performed with polyclonal goat antimouse IgG2a for antimouse Ig and antimouse IgG2a antibodies (GAM/IgG2a/7S, Nordic Immunological Laboratories, Eindhoven, The Netherlands) and goat antirat IgG2b for antirat Ig and antirat IgG2b antibodies (GARa/IgG2b/7S, Nordic Immunological Laboratories, Eindhoven, The Netherlands).

The first 20 evaluable patients were tested for HAMAs and HARAs by in-house assays. Both HAMAs and HARAs were induced by catumaxomab therapy and ADAs were directed against both the mouse and rat components of the catumaxomab molecule. Thus, for the purposes of this *post hoc* analysis, the HAMA assay was considered to be representative for ADAs, including HARAs.

### Correlation between HAMA status and clinical outcome

The humoral response was measured by induction of HAMAs. HAMA-positive and HAMA-negative patients in the catumaxomab group and patients in the control group were analyzed separately for all three clinical outcome measures and compared with each other. The HAMA status used for the analysis was that measured at Visit 6, *i.e*., 8 days after the fourth infusion of catumaxomab. This timepoint was selected for the following reasons: (1) patients' immune systems had sufficient time to develop a response (before the fourth infusion only a few patients show a response) and (2) at this timepoint, data were available for the majority of patients. Clinical outcome was measured as OS, puncture-free survival and time to next therapeutic puncture. The potential correlation between the immune response 8 days after completion of catumaxomab treatment and clinical outcome in the randomized Phase II/III pivotal study was investigated in a *post hoc* analysis. The potential correlation was analyzed for the overall population, patients with ovarian and nonovarian cancer and patients with gastric cancer, the largest subgroup of patients in the nonovarian cancer group.

### Statistics

All statistical tests were two-sided at the 5% significance level and were explorative in nature; therefore no adjustment for multiple testing was performed. Time-to event parameters (OS, puncture-free survival and time to first puncture) were compared between the treatment groups using the log-rank test and HRs, including 95% confidence intervals (CIs). Compliance rates were compared using Fisher's exact test.

Statistical analyses were performed for three analysis sets: the intent-to-treat (ITT) population (all randomized patients); the safety population (all patients who received at least one dose of catumaxomab or who were randomized to the control group) and the per-protocol population (all patients in the safety population who had no major protocol violations, defined as receiving less than three doses of catumaxomab, puncture performed although criteria were not fulfilled and violation of inclusion and/or exclusion criteria). The HAMA analyses were based on patients who were randomized to catumaxomab and for whom HAMA measurements were available at Visit 6 (8 days after the fourth infusion). To minimize a potential selection bias, only patients randomized to the control group who attended Visit 6 (8 days after Day 0, before a potential crossover) were considered for the three-group comparison (HAMA-positive catumaxomab patients, HAMA-negative catumaxomab patients and control patients). For the primary endpoint (puncture-free survival), any patient lost to follow-up was censored at the time of their last visit. In addition, for the OS analysis, patients randomized to the control group who then crossed over to catumaxomab treatment were censored at the time of cross-over, *i.e*., OS data collected after the crossover was not considered.

## Results

### HAMA status

Before treatment with catumaxomab, serum samples from almost all patients (>95%) were HAMA negative. Of 170 patients randomized to the catumaxomab group, 157 patients received at least one catumaxomab infusion. Of these, 112 patients (66% of the patients randomized to catumaxomab, 71% of the patients who received at least one catumaxomab infusion) were evaluable for HAMA assessment. In the control group, 50 of 88 patients (57%) were evaluable at visit 6. Before the fourth infusion, most patients [91% (59/65) ovarian cancer and 98% (58/59) nonovarian cancer] were still HAMA negative. Only 6% (7/124) of patients who received at least one catumaxomab infusion developed HAMA values in serum before the fourth infusion. The percentage of HAMA-positive patients 8 and 28 days after the fourth catumaxomab infusion and at the puncture visit was 76, 94 and 84%, respectively.

Eight days after the fourth infusion, 76% (85/112) of the evaluable catumaxomab patients were HAMA positive. This increased to 94% of 70 evaluable patients 28 days after the fourth infusion then decreased to 84% of 45 evaluable patients at the puncture visit. Table 1 shows the number of HAMA-positive and HAMA-negative catumaxomab patients in the overall and subgroup populations 8 days after the fourth infusion. The compliance rate (*i.e*., patients receiving all four infusions of catumaxomab) in this selected patient group was 95% compared with 83% for all patients in the safety population (Tables 1 and 2).

**Table 1:** HAMA status and compliance in relation to HAMA status of catumaxomab patients 8 days after the fourth infusion (Visit 6)

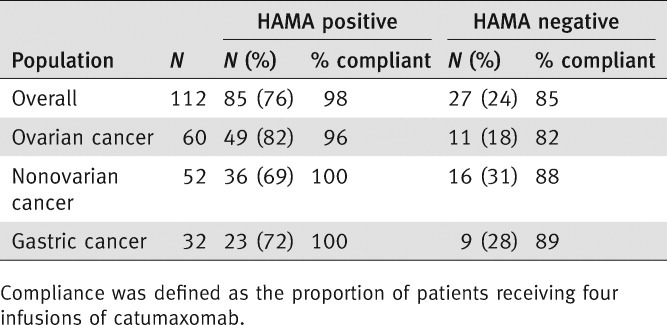

**Table 2:** Compliance rates

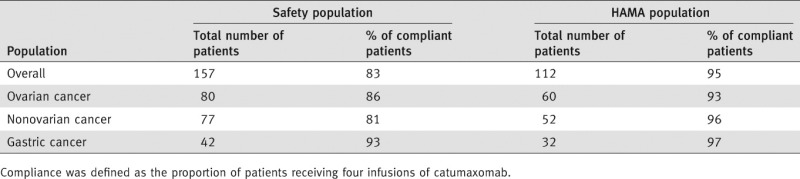

In the overall population and each subgroup population, the compliance rate of HAMA-positive patients was higher than HAMA-negative patients. In the overall population, the difference in the compliance rate between HAMA-positive (98%) and HAMA-negative patients (85%) was statistically significant (*p* = 0.0292). The median HAMA concentration in serum was 1241 μg/L 8 days after the fourth infusion (range 0.0–500,000 μg/L). The HAMA concentration was not predictive for clinical efficacy. In addition, there was no relationship between the occurrence of HAMAs and the pattern of adverse events reported after the last infusion.

### Clinical outcome

**Figure 1:**
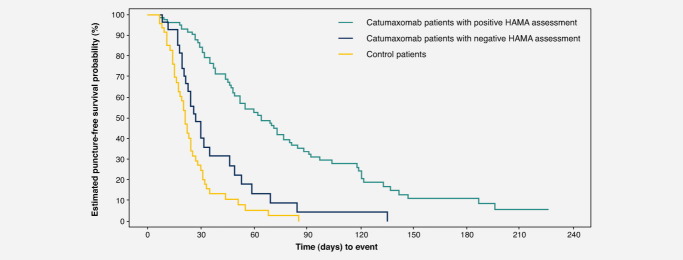
Puncture-free survival in the overall population.

**Figure 2:**
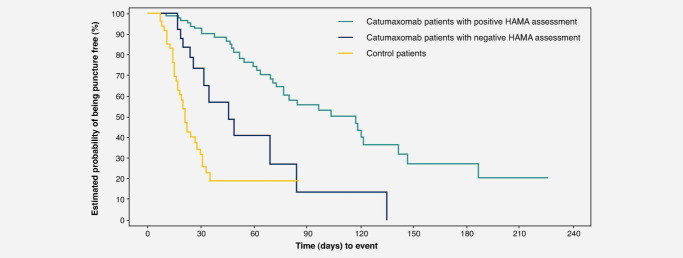
Time to first therapeutic puncture in the overall population.

At visit 6, catumaxomab treatment resulted in significantly greater clinical benefit for HAMA-positive *vs*. HAMA-negative patients. Median puncture-free survival was 64 days in HAMA-positive patients *vs*. 27 days in HAMA-negative patients (*p* < 0.0001; HR 0.330) (Fig. 1). In patients with ovarian cancer, median puncture-free survival was 64 *vs*. 30 days (*p* = 0.0016; HR 0.306). In patients with nonovarian cancer, median puncture-free survival was 67 *vs*. 25 days (*p* = 0.0010; HR 0.366). In patients with gastric cancer, median puncture-free survival was 70 *vs*. 27 days (*p* < 0.0001; HR 0.166). Median time to next therapeutic puncture was 104 days in HAMA-positive patients *vs*. 46 days in HAMA-negative patients (*p* = 0.0002; HR 0.307) (Fig. 2). In patients with ovarian cancer, median time to next therapeutic puncture was 104 *vs*. 32 days (*p* = 0.0055; HR 0.273). In patients with nonovarian cancer, median time to next therapeutic puncture was 118 *vs*. 49 days (*p* = 0.0138; HR 0.321). In patients with gastric cancer, median time to next therapeutic puncture was 122 *vs*. 49 days (*p* = 0.0378; HR 0.161).

OS was increased with catumaxomab *vs*. paracentesis alone: in the ITT population, the 1-year OS rate was 10.9% *vs*. 3.4% (*p* = 0.0846; HR 0.723); in the safety population, the difference was statistically significant (1-year OS rates of 11.4% *vs*. 3.4%; *p* = 0.0242; HR 0.654); while in the per-protocol population, the difference was more pronounced (12.7% *vs*. 3.5%; *p* = 0.0085; HR 0.597) (Table 3). HAMA-positive patients showed a significantly better OS compared with HAMA-negative patients in the overall population and in patients with ovarian, nonovarian and gastric cancer (Table 4). Median OS was 129 days in HAMA-positive patients *vs*. 64 days in HAMA-negative patients (*p* = 0.0003; HR 0.433). In patients with ovarian cancer, median OS was 163 *vs*. 82 days (*p* = 0.0123; HR 0.407). In patients with nonovarian cancer, median OS was 99 *vs*. 47 days (*p* = 0.0451; HR 0.543). In patients with gastric cancer, median OS was 111 *vs*. 38 days (*p* = 0.0260; HR 0.412).

**Table 3:** Overall survival

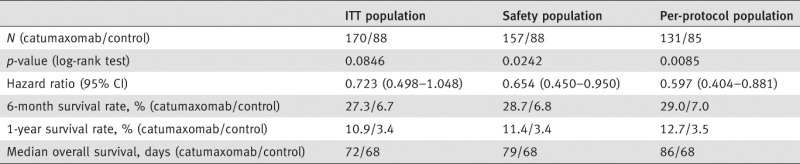

**Table 4:** Correlation between clinical outcome and humoral response

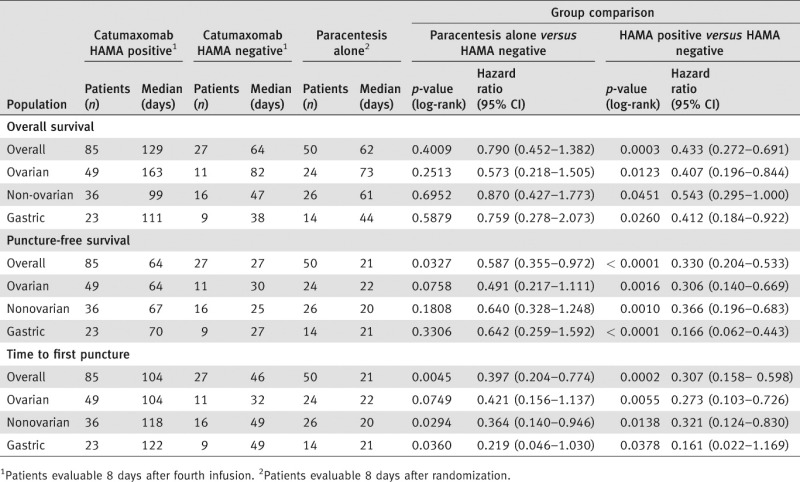

## Discussion

The results of this analysis demonstrate that there was a strong correlation between humoral response to catumaxomab and clinical outcome in the pivotal Phase II/III study of patients with malignant ascites: patients who developed HAMAs after catumaxomab treatment showed significant improvement in clinical outcome, as measured by puncture-free survival, time to next puncture and OS, compared with HAMA-negative patients in the overall population and in patients with ovarian, nonovarian and gastric cancer. As noted in the methods section, the HAMA assay was considered to be representative for ADAs including HARAs and thus indicates the ADA response to catumaxomab treatment. Interestingly, median OS in patients who developed an immune response to catumaxomab was more than double that in catumaxomab patients who did not develop an immune response or those treated with paracentesis alone. The results indicate that patients with a functional immune system are particularly responsive to catumaxomab treatment, inducing a very efficient humoral reaction, resulting in marked improvement in OS. The antidrug response against catumaxomab treatment can be regarded as a surrogate marker for clinical activity and may serve as an indicator of clinical outcome.

In the overall population, the difference in the compliance rate (all four infusions received) between HAMA-positive (98%) and HAMA-negative patients (85%) was statistically significant (*p* = 0.0292, Fisher's exact test). This may reflect the importance of receiving all four infusions to achieve an optimal antitumor response and may explain the difference in clinical outcome between these two patients groups. These results are in agreement with results of a Phase IIa study of patients with platinum-resistant epithelial ovarian cancer who received either low- or high-dose catumaxomab.^18^ In this study, a clinical benefit was achieved in 27% of patients in the high-dose group (one patient with a partial response and five patients with stable disease) *vs*. 9% of patients in the low-dose group (two patients with stable disease). The clinical efficacy of catumaxomab may therefore be dependent on the dose used, as shown by Belau *et al*.,^18^ and/or the development of HAMAs, as shown in our study, which only occurs to a significant degree after the fourth infusion and may therefore be linked to patient compliance. Indeed, when we consider OS data for the safety population (*i.e*., patients who received at least one catumaxomab infusion) and the per-protocol population (*i.e*., patients who received at least three infusions), clinical outcome seems to improve further with increased doses, which in turn is associated with an increased HAMA response.

Potential confounding factors that could have affected the result are the treatment received during the poststudy period, the amount of prior antineoplastic medication and the Karnofsky status of the patients. No association was found between HAMA status or clinical outcome and the number of previous lines of chemotherapy. In the overall population, there was a trend for HAMA-positive status to correlate with a better Karnofsky status, *i.e*., the median Karnofsky status for HAMA-positive patients was 80, compared with a median of 70 for HAMA-negative patients (*p* = 0.0419, Wilcoxon-test). However, it is unlikely that any of these potential confounding factors influenced the correlation between HAMA status and clinical outcome.

In the pivotal study, a single treatment cycle consisting of four ascending catumaxomab doses was investigated. A repeated treatment cycle is assumed to further enhance the efficacy of catumaxomab. However, the development of ADAs could potentially interfere with the efficacy of a repeated cycle of catumaxomab due to interception of the antibody. In a case study, a heavily pretreated patient with breast cancer received a repeated cycle of catumaxomab, which resulted in a clear reduction in the number of tumor cells and an increase in the number of immune accessory cells in ascites samples, corresponding to a prolonged paracentesis-free interval.^19^ These effects were observed despite an increase in HAMA concentrations in the ascites and serum (from 340 to 587,983 ng/mL and from 1627 to 783,893 ng/mL, respectively, after the fourth infusion). It therefore appears that the development of ADAs does not automatically affect the clinical efficacy of catumaxomab. On the basis of this case, an open-label, single-arm, Phase II study (SECIMAS) to investigate the efficacy and safety of a repeated cycle of catumaxomab has been initiated.^19^

To our knowledge, this is the first pivotal clinical study in which a strong correlation between humoral response to an approved mAb and clinical outcome has been demonstrated. The correlation between HAMA status and clinical outcome in our study is similar to that reported in other smaller, nonpivotal clinical studies of murine mAbs.^12^–^15^, ^17^, ^20^–^22^ In a study of 34 patients with stage 4 neuroblastoma treated with the anti-G(D2) monoclonal antibody 3F8 at the end of chemotherapy, a transient HAMA response was associated with significantly longer survival.^13^ In addition, long-term OS and progression-free survival correlated significantly with the anti-G (D2) response at 6 months and with the anti-antiidiotypic antibody response at 6 and 14 months.^14^ Survival was significantly longer (103 *vs*. 61 weeks) in patients who developed high HAMA titers (≥5 μg/mL) *vs*. those with low titers (<5 μg/mL) at 16 weeks in a study that assessed the relationship between HAMA status and survival in 39 patients with relapsed B-cell malignancies treated with the mouse antilymphoma mAb Lym-1.^12^

By definition, the immune response to the administration of mAbs, resulting in the production of HAMAs, requires a functional immune system, despite the fact that patients may have been heavily pretreated. In our study, patients had received 0–10 previous lines of chemotherapy. The immune response involves a series of processes, including cellular antigen processing, T-cell activation and the differentiation of B-cells into Ab-secreting plasma cells that produce HAMAs, with HAMA production occurring after a minimum of 7 days following mAb administration.^11^ Whether or not HAMAs develop in response to mAb administration may be dependent on a number of factors, including interindividual immunogenetic differences,^23^ tumor-induced immunosuppression,^9^ and the patient's initial immune status.^15^ The beneficial humoral effects and prolonged survival associated with the development of ADAs indicate that they may be surrogate markers for mAb efficacy.

The beneficial effects of an ADA response could be due to a number of mechanisms. One potential mechanism is the network hypothesis, which postulates that the antiidiotype antibody is itself immunogenic and induces a humoral immune response by the formation of anti-antiidiotypic antibodies. The antiidiotype, which represents an internal image of the antigen, is thought to mimic an epitope of the antigen the initial antibody binds to (the antiidiotype), so the immune response is directed against the primary target in addition to the antiidiotypic antibody. Complexes of the murine therapeutic antibody and the human antiidiotype are internalized and presented by antigen-presenting cells, potentially stimulating T-cells and resulting in an antitumor response. Such antiidiotypic networks have already been shown to be a promising therapeutic approach.^21^, ^22^ Antibody-dependent cellular cytotoxicity may result in direct tumor destruction, inducing tumor-antigen-specific T-cell responses, with host antitumor immunity occurring by the production of tumor-directed cytotoxic T-cells, antibodies, or both.^24^ Ströhlein *et al*. showed that treatment with catumaxomab and the trAb ertumaxomab induced an increase in autologous tumor-reactive T-cells in five of nine patients with peritoneal carcinomatosis.^7^ T-cell activation was also demonstrated by increased serum levels of soluble interleukin-2 receptor. A second potential mechanism is the direct induction of a cellular immune response by the murine antibody through its major histocompatibility complex presentation by antigen-presenting cells. The processed mouse antibody presented on the tumor cell would serve as a new artificial tumor-associated antigen that could be recognized by cytotoxic T cells. A cellular immune response would explain the efficacy of an antibody therapy even after the formation of neutralizing ADAs. Given the potential benefits of ADAs, the utility and value of human or humanized antibodies *vs*. animal-derived antibodies has been questioned and should be decided after a case by case evaluation.^25^

According to the definition of HAMA status, only patients who reached Visit 6 (8 days after the fourth infusion) and for whom HAMA measurements were performed were considered. Thus, the observed catumaxomab patient population decreased from 170 randomized patients to 157 patients who received at least one catumaxomab infusion and to 112 evaluable patients, as 34% of the randomized patients (29% of the treated patients) who dropped out or died before this timepoint were not considered. To account for this selection, only those control patients who reached Visit 6 were considered for the comparison. As a result, 66% of patients in the catumaxomab group and 57% of those in the control group were evaluable. To estimate the size of the selection bias, both the total and evaluable catumaxomab patient groups were compared for the three clinical outcome parameters. In summary, all comparisons between catumaxomab patients and control patients were subject to bias. However, this bias was minimized by maximizing the similarity between the patient groups, given these limitations.

Our results show that OS was significantly prolonged in patients treated with catumaxomab and that this benefit appears to increase with the catumaxomab dose. Patients with a functional immune system were particularly responsive to catumaxomab treatment. Thus, the use of catumaxomab at an earlier stage of disease could result in even more beneficial effects. The short treatment duration of 10 days would allow for integration into existing treatment regimens. *In vitro* data indicate synergy of catumaxomab with chemotherapeutic drugs.^26^ Clinical studies investigating combinations of catumaxomab with chemotherapies are in development. In this study, the HAMA response appeared to be associated with patients' clinical status and prognosis, as shown by the trend for HAMA-positive status to correlate with a better Karnofsky status. Thus, the development of HAMAs may serve as a biomarker for response and the search for a predictive marker is ongoing. In addition, HAMAs induced by catumaxomab may be pharmacologically active. To date, there is no evidence for any safety concerns with the HAMAs induced by catumaxomab, which is in agreement with the results of human antiglobulin antibody responses to other monoclonal antibodies.^9^–^11^ The potential pharmacological activity of HAMAs requires further investigation. As the induction of a HAMA response is a surrogate marker for a functional immune system, the use of catumaxomab at a less advanced disease stage could potentially result in greater clinical benefit. This will be investigated in future clinical studies.

## Abbreviations:

ADA: antidrug antibodies;
BSA: bovine serum albumin
CI: confidence interval
ELISA: enzyme-linked immunosorbent assays
EpCAM: epithelial cell-adhesion molecule
HAMA: human antimouse antibody
HARA: human antirat antibody
HR: hazard ratio
i.p.: intraperitoneal
Ig: immunoglobulin
ITT: intent-to-treat
mAb: monoclonal antibody
OS: overall survival
trAb: trifunctional antibody
